# The Effect of the Extraction Medium (A Natural Deep Eutectic Solvent-Derived System vs. Ethanol) on the Properties of Electrospun PVA Fibers Containing *Quercus robur* Extracts

**DOI:** 10.3390/ma19091730

**Published:** 2026-04-24

**Authors:** Julia Wnękowicz, Daniel Szopa, Paulina Wróbel, Julia Zwolińska, Maciej Kaniewski, Jacek Chęcmanowski, Anna Witek-Krowiak

**Affiliations:** 1 Faculty of Chemistry, Wroclaw University of Science and Technology, 50-370 Wroclaw, Poland; 265614@student.pwr.edu.pl; 2 Department of Engineering and Technology of Chemical Processes, Faculty of Chemistry, Wrocław University of Science and Technology, Gdańska 7/9, 50-344 Wrocław, Poland; daniel.szopa@pwr.edu.pl (D.S.); julia.zwolinska@pwr.edu.pl (J.Z.); maciej.kaniewski@pwr.edu.pl (M.K.); 3 Department of Advanced Material Technologies, Faculty of Chemistry, Wroclaw University of Science and Technology, Smoluchowskiego 25, 50-370 Wroclaw, Poland; jacek.checmanowski@pwr.edu.pl

**Keywords:** electrospinning, natural deep eutectic solvents, *Quercus robur*, poly(vinyl alcohol), polyphenols

## Abstract

This study examined how the extraction medium used to obtain *Quercus robur* extracts influenced the properties of electrospun poly(vinyl alcohol) (PVA) mats intended for potential active packaging applications. Extracts prepared with 50% ethanol and with a choline chloride:lactic acid:water system were incorporated into PVA spinning solutions, and their effects on solution properties, fiber morphology, thermal behavior, crosslinking response, and polyphenol release were evaluated. The type of extraction medium affected both the electrospinning process and the structure of the resulting materials. Ethanol-derived extracts reduced solution viscosity and promoted the formation of thinner fibers, whereas systems containing the choline chloride:lactic acid:water-derived extract showed higher conductivity and lower electrospinning stability. Crosslinking with tannic acid in water led to the collapse of the fibrous structure, while ethanolic tannic acid treatment preserved the nanofibrous morphology more effectively. FTIR analysis indicated differences in intermolecular interactions within the polymer matrix, consistent with the observed changes in structural stability and release behavior. Thermal analysis showed that ethanol-derived extracts lowered the thermal stability of the PVA matrix, whereas the choline chloride:lactic acid:water-derived system altered the degradation pathway and increased the amount of solid residue formed during heating. Release studies demonstrated a rapid burst release for ethanol-based mats and a more sustained release profile for mats containing the choline chloride:lactic acid:water-derived extract. Selected extract-containing and ethanol–tannic acid-crosslinked mats also showed antibacterial activity against *Staphylococcus aureus*. The results showed that the extraction medium significantly affected polymer–extract interactions and the functional properties of electrospun PVA mats. At the same time, the conclusions refer specifically to the tested solvent systems, and broader generalization to other natural deep eutectic solvent-type formulations requires further comparative studies.

## 1. Introduction

In response to the growing demand for food with extended shelf life and high-quality, active packaging systems are gaining increasing importance. Conventional packaging primarily serves a passive protective role, whereas active packaging materials dynamically interact with food, for example, by absorbing or releasing compounds to prevent spoilage [1]. Particularly promising is the use of natural bioactive substances, such as plant-derived polyphenols, which exhibit antioxidant and antimicrobial activity, thereby helping preserve food quality and extend shelf life [2]. At the same time, incorporating such compounds into polymer matrices may significantly influence not only their biological function but also the material’s physicochemical behavior.

Among the emerging fabrication approaches for such packaging materials, electrospinning is considered one of the most promising techniques [3]. This method enables the production of ultralight nanofibrous mats with very high specific surface area and porosity [4]. Such structures provide intense contact with the surrounding environment, which may enhance the release of active compounds and their interaction with packaged food. An additional advantage of electrospinning is the ability to process at relatively low temperatures, enabling the incorporation of thermolabile compounds without degradation. Poly(vinyl alcohol) (PVA) is widely used in this context due to its biocompatibility, biodegradability, and ease of processing [5]. However, its high hydrophilicity leads to rapid swelling or dissolution in aqueous environments, which may limit structural stability and result in uncontrolled release of incorporated compounds.

Numerous studies have demonstrated that incorporating plant extracts rich in polyphenols into electrospun PVA systems enables the development of materials with functional properties relevant to food packaging applications. For example, electrospun PVA mats containing tea polyphenols have been shown to provide controlled release and prolonged protective activity [6]. Polyphenols embedded in PVA matrices may interact with polymer chains through hydrogen bonding, which can influence both thermal stability and release behavior [7]. Other studies confirm that such systems may improve the microbiological stability of food products and extend shelf life [8]. These findings highlight the dual role of plant-derived additives, acting not only as bioactive agents but also as modifiers of polymer structure and material performance.

Acorn extracts (*Quercus* spp.) represent a promising yet underexplored source of polyphenols for such applications. Acorns are rich in phenolic compounds, including phenolic acids, flavonoids, and tannins, which are associated with antioxidant and antimicrobial activity [9]. Their biological activity has been confirmed in several studies, including the inhibition of pathogenic bacteria and biofilm formation [10]. Despite this potential, acorns remain an underutilized biomass resource. Their application in packaging materials is consistent with current trends in sustainable development and the valorization of natural waste streams. The use of acorn extracts in materials science remains limited, and existing studies have focused primarily on biomedical applications [11]. Recent work on alginate-based films has shown that acorn-derived polyphenols obtained using different extraction media can affect polymer structure and functional performance, including intermolecular interactions, thermal behavior, and release properties [12]. However, such effects should be interpreted with caution, because dilution and the presence of additional solvents may disturb the original eutectic organization. As a result, these systems may behave more like mixtures of solvated ionic and polar components than as fully preserved natural deep eutectic solvent (NADES).

Despite these findings, the application of acorn extracts in electrospun nanofibrous systems remains largely unexplored. In particular, the combined effects of the extraction medium, polymer–extract interactions, and crosslinking strategy on the structure, stability, and release performance of electrospun PVA mats have not been systematically investigated. Due to their high surface area and sensitivity to post-treatment, such nanofibrous systems may be particularly susceptible to changes in structural integrity and functional performance.

Therefore, the aim of this study was to investigate how the extraction medium used for obtaining *Quercus robur* extracts affected the properties of electrospun PVA mats. Special attention was given to the relationships between solution properties, fiber morphology, intermolecular interactions within the polymer matrix, thermal stability, crosslinking response, and the release behavior of polyphenolic compounds in food-simulating media.

**Figure 1:**
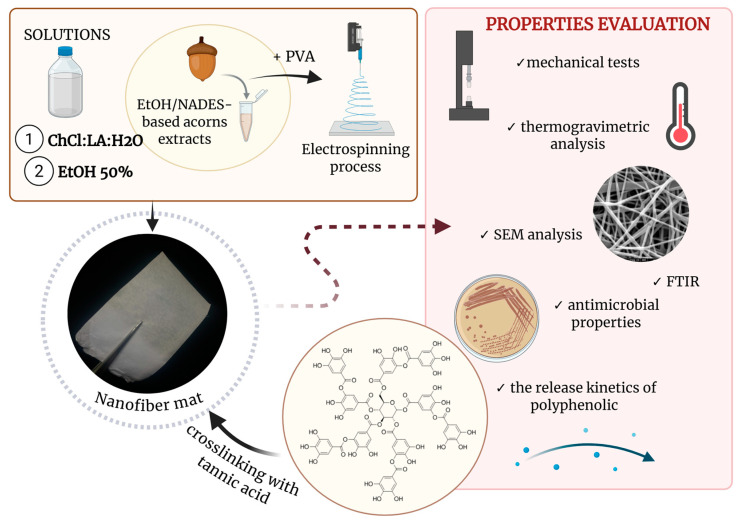
Overall concept of research. Created in BioRender. Witek-Krowiak, A. (2026) https://BioRender.com/7lsuz7d (accessed on 30 March 2026).

The overall concept and workflow of the study are presented in Figure 1.

## 2. Materials and Methods

### 2.1. Materials

All chemicals and biological materials used throughout the study were of commercial origin and applied as received. Acorn flour was obtained from Dary Natury (Grodzisk, Poland). Sigma-Aldrich (St. Louis, MO, USA) supplied choline chloride (ChCl), poly(vinyl alcohol) (PVA, 130 kDa), sodium carbonate, and gallic acid monohydrate. L-Lactic acid (LA, 80%) together with the Folin–Ciocalteu reagent were obtained from Chempur (Piekary Śląskie, Poland). Ethanol (99.8%), boric acid (BA), and tannic acid (TA) were purchased from Stanlab (Lublin, Poland), POCH (Gliwice, Poland), and ARCHEM (Zakroć, Poland), respectively. The culture media Luria–Bertani (LB) broth and LB agar were obtained from A&A Biotechnology (Gdańsk, Poland). The antimicrobial tests were carried out using *E. coli* ATCC 10536, *P. aeruginosa* ATCC 15442, and *S. aureus* ATCC 25923 strains acquired from ATCC (Manassas, VA, USA).

### 2.2. Polyphenol Extraction

The solvent systems used in this study were selected based on preliminary solvent-screening experiments performed for the extraction of polyphenolic compounds from *Quercus robur*. Based on these results, 50% ethanol and the ChCl:LA:water-derived system were chosen for further investigation in the electrospinning study. The NADES components (ChCl:LA 1:2) were combined and mixed at 60 °C until a clear and homogeneous liquid was formed. After preparation of the initial ChCl:LA system, water was added prior to extraction, so the final extraction medium should be understood as a ChCl:LA:water-derived multicomponent system rather than as a fully preserved classical NADES. Subsequently, the prepared system was mixed with water to achieve a mass proportion of 0.75:0.25 (ChCl:LA:water/water). Extractions with ChCl:LA:water and 50% EtOH were performed at 60 °C for 60 min, using a solid-to-solvent ratio of 1:10 (*w*/*w*) under constant stirring at 300 rpm. Following the extraction process, the samples were centrifuged at 6000 rpm for 10 min using a FRONTIER FC5706 centrifuge (OHAUS, Nänikon-Greifensee, Switzerland). The obtained extracts were subsequently filtered and stored at 4 °C until further analysis.

### 2.3. Determination of Total Phenolic Content and DPPH Inhibition

The total phenolic content (TPC) of the obtained extracts was quantified using a modified Folin–Ciocalteu procedure [13]. Briefly, 100 µL of appropriately diluted extracts was combined with 6 mL of distilled water and 500 µL of Folin–Ciocalteu’s reagent 10 mL in a 10 mL volumetric flask. After an initial reaction period of 3 min, the mixture was combined with 1.5 mL of saturated sodium carbonate solution and the volume was adjusted to the mark with distilled water, followed by thorough mixing. The solutions were thoroughly homogenized and incubated at 40 °C for 30 min in water bath. After incubation, absorbance was measured at 765 nm using a S1020 UV-Vis spectrophotometer (Techcomp, Hong Kong, China). Gallic acid standard solutions (0–0.5 mg/mL) were prepared in distilled water to construct a calibration curve. Total phenolic content was expressed as milligrams of gallic acid equivalents per gram of dry weight (mg GAE/g d.w.). Reagent blanks were included in all measurements to correct for background absorbance. TPC in the release study, due to low polyphenol concentrations, was determined using the method described in [6].

The antioxidant activity of the extracts was determined by the DPPH inhibition method with slight modification [14]. For this purpose, a DPPH solution in methanol at 0.0625 mg/mL was prepared. To the extracts in a volume of 100 μL, 4 mL each of the DPPH solution was added. The prepared solution, with no addition of extract, served as the control sample. The mixtures were kept in the dark for 30 min, after which absorbance was measured at 517 nm. The percentage of DPPH inhibition was calculated using Equation (1):

DPPH inhibition (%) = (Ac − As)/Ac × 100 (1)

where Ac represents the absorbance of the control and As denotes the absorbance of the samples with extract addition.

### 2.4. Electrospinning Solutions Preparation

To prepare the polymer matrix, PVA was dissolved in distilled water at a concentration of 14 wt%. The dissolution process involved an initial stirring step at room temperature for 30 min, followed by heating to 100 °C with continuous magnetic stirring for 2 h. Once the polymer had completely dissolved, the extracts were added in the amounts specified in Table 1. The resulting mixtures were stirred at room temperature until homogeneous solutions were obtained.

### 2.5. Viscosity Measurements

The viscosity characteristics of the solutions were determined by rotational rheometry using a ViscoQC 100 instrument (Anton Paar, Graz, Austria). The device was operated with RH2 or RH3 spindles, depending on the sample type, and the measurements were conducted over a speed interval of 10–60 rpm. Among the tested formulations, only the Et3 sample required the RH2 spindle, whereas the other solutions were assessed with the RH3 spindle. The viscosity measurements were performed to compare the spinning solutions within the tested rotational speed range and did not include a full viscoelastic characterization of the systems.

### 2.6. Solution Conductivity Testing

An HI-98304 conductivity meter manufactured by Hanna Instruments (Olsztyn, Poland) was used to assess the conductivity of the electrospinning solutions under room-temperature conditions.

### 2.7. Electrospinning

Electrospinning was employed to produce nanofibers from CS, Et1, Et2, Et3, DES1, DES2, DES3, DES4, and DES5 solutions using a Professional Lab Device electrospinning apparatus (DOXA Microfluids, Malaga, Spain). In all experiments, the solution was delivered through a syringe with a 20-gauge needle. The collector rotation speed was maintained at 100 rpm, and the working distance between the needle tip and the collector was set to 10 cm. Since the individual formulations required different processing conditions, the applied voltage and solution feed rate were selected separately for each case to maintain a stable spinning regime, as presented in Table 2. Owing to differences in feed rate, the duration of electrospinning was also varied to obtain mats of similar thickness and comparable morphology. All electrospinning runs were performed at room temperature, with the relative humidity kept within the range of 30–40%.

### 2.8. Scanning Electron Microscopy

Scanning electron microscopy was used to evaluate the morphology of the obtained nanofiber mats. The observations were carried out with a Quanta 250 microscope (FEI, Hillsboro, OR, USA) fitted with a secondary electron detector. Before SEM analysis, the samples were sputter-coated with gold (~20 nm, 99.99% purity). During imaging, the working distance was maintained at around 10 mm and the chamber pressure remained below 10^−4^ Pa. The average fiber diameter was assessed manually with ImageJ 1.54g software (National Institutes of Health, Bethesda, MD, USA) by analyzing 100 randomly chosen fibers.

### 2.9. Crosslinking of Electrospun Mats with TA Aqueous Solution

For crosslinking, a 3 wt% aqueous TA solution was prepared. The TA solution was stirred at ambient temperature until a homogeneous solution was obtained. The mats were crosslinked by spraying them with TA solution from 10 cm, followed by air-drying under ambient conditions. The amount of solution applied was controlled to achieve a loading of approximately 4.5 mg/cm^2^ of mat.

### 2.10. Alternative Crosslinking Method

An alternative crosslinking method using ethanolic TA solutions was applied. The electrospun mats were sprayed with 4.5 mg/cm^2^ TA solutions prepared in ethanol at concentrations of 3 wt% and 8 wt%, with a spraying distance of approximately 10 cm to ensure uniform surface coverage. Spraying was continued until the entire surface of the mat was visibly and uniformly covered with the crosslinking agent. Following the application, selected samples were subjected to thermal treatment at 155 °C for 10 min, as reported for tannic acid-crosslinked PVA nanofibrous membranes [15]. As a result, four crosslinking variants were obtained: TA applied in ethanol at two concentrations (3 wt% and 8 wt%), each with and without subsequent heat treatment. Samples crosslinked with 3 wt% aqueous TA are denoted as 3%TA-W, while ethanolic systems are labeled as 3%TA-E and 3%TA-E-H (with thermal treatment). The same nomenclature is applied for 8 wt% TA (8%TA-E, 8%TA-E-H).

### 2.11. Mechanical Testing

Tensile behavior of the nanofiber mats was characterized using an INSTRON 5966 universal testing system (INSTRON, Norwood, MA, USA). Rectangular specimens with dimensions of approximately 40 mm × 10 mm were tested at a crosshead speed of 10 mm/min, and the resulting stress–strain profiles were used to determine tensile strength and elongation at break. Due to the delicate nature of the electrospun materials, the samples were mounted in paper frames measuring 50 mm × 60 mm and containing a 30 mm × 30 mm inner opening. This procedure was based on a literature method with minor modifications [12]. The ends of each specimen were secured to the frame with double-sided tape, and the lateral parts of the frame were cut immediately before testing to release the sample. Ten specimens of each sample were tested.

### 2.12. FTIR

The chemical structure of the mats was investigated by FTIR spectroscopy using an IRAffinity-1S spectrometer (Shimadzu, Kyoto, Japan) fitted with an ATR accessory. Spectra were recorded between 4000 and 400 cm^−1^, at a spectral resolution of 4 cm^−1^, with 256 scans collected for each sample.

### 2.13. TGA

Thermogravimetric analysis was performed with a STA 449 F3 instrument connected to an Aëolos QMS 403 C mass spectrometer (NETZSCH-Gerätebau GmbH, Selb, Germany). For each run, about 5 mg of sample was weighed into an open alumina crucible (volume 0.085 cm^3^). The experiments were conducted in a synthetic air atmosphere supplied at 30 mL/min, while the temperature program involved heating up to 1100 °C at 10 K min^−1^. Under these conditions, the effective temperature of the sample reached approximately 1040 °C. Before analysis, baseline correction was performed using an empty crucible to account for thermal contributions of the sample holder. Data collection and analysis were carried out using NETZSCH Proteus 8.0.3. software.

### 2.14. Polyphenol Release

The release kinetics of polyphenolic compounds from electrospun mats containing Et3 and DES2 extracts were evaluated for samples crosslinked using three different methods. The study was conducted in four food stimulants: water, 10 wt% ethanol, 50 wt% ethanol, and 3 wt% acetic acid solution at room temperature with gentle shaking. For each experiment, 5 mg of the electrospun mat was immersed in 5 mL of the respective medium and maintained under controlled conditions. Aliquots of 100 µL were taken at selected time intervals (20, 40, 60, 90 min, and after 3, 6, and 24 h) for subsequent analysis. After each sampling step, an equal volume of fresh medium was added in order to keep the total volume and experimental conditions unchanged.

To quantitatively describe the release kinetics of polyphenols from the electrospun mats, the experimental data were fitted using the Weibull model, which is widely applied for the analysis of release processes from polymeric systems [12]. The model is expressed as follows:

(2) $$\frac{M_{t}}{M_{\infty }}=1-exp\left({-\left(\frac{t}{a}\right)}^{b}\right)$$

where $M_{t}$ corresponds to the quantity of polyphenols released after time t, and $M_{\infty }$ is the maximum amount released at infinite time. The parameter a reflects the time-related scale of the process, where b is the shape parameter associated with the release mechanism. In addition, the release data were analyzed using the Korsmeyer–Peppas model and the Peppas–Sahlin model in order to provide further insight into the underlying release mechanisms [16]. The Korsmeyer–Peppas model is given by:

(3) $$\frac{M_{t}}{M_{\infty }}=k\;t^{n}$$

where k is the kinetic constant and n is the release exponent indicative of the transport mechanism. This model was applied to the initial stage of release ($M_{t}/M_{\infty }\leq 0.6$), in accordance with its theoretical assumptions. The Peppas–Sahlin model, which accounts for the combined effects of diffusion and polymer relaxation, is expressed as:

(4) $$\frac{M_{t}}{M_{\infty }}=k_{1}t^{m}+k_{2}t^{2m}$$

where $k_{1}\;$ corresponds to the contribution of Fickian diffusion, $k_{2}$ represents the contribution of polymer relaxation (swelling or structural rearrangement), and m is the diffusional exponent. The modeling and data analysis were carried out using Python 3, with the SciPy library for numerical analysis and curve fitting.

### 2.15. Antibacterial Properties

The antibacterial properties of the samples were evaluated according to the method of Szopa et al. [12], with minor modifications. Briefly, bacterial strains were propagated in LB broth at 37 °C for 24 h with shaking at 160 rpm. After cultivation, the cells were collected by centrifugation at 7000 rpm for 5 min, washed twice with sterile distilled water, and diluted to 1 × 10^6^ CFU/mL based on OD600 measurements. For the assay, 100 μL of each suspension was spread onto agar plates and left to dry. To assess CS, Et3 and DES2 activity, discs (approximately 17.5 mm in diameter) were positioned centrally on the inoculated agar. The plates were incubated for 24 h at 37 °C and the resulting inhibition zones were measured. All measurements were performed in triplicate and reported as average values with standard deviations.

## 3. Results

### 3.1. Determination of Total Phenolic Content and DPPH Inhibition

The extracts incorporated into the electrospinning solutions were first characterized in terms of their total phenolic content. The ethanol extract exhibited a TPC of 40.46 ± 2.12 mg GAE/g, whereas the ChCl:LA:water-derived extract showed a higher TPC of 68.70 ± 1.83 mg GAE/g. The higher extraction efficiency may be attributed to its enhanced ability to solubilize polyphenolic compounds, driven by strong hydrogen-bond interactions between the solvent components and phenolic molecules [17]. The DPPH inhibition was also determined for the extracts used in the spinning solutions. The ChCl:LA:water-derived extract exhibited a DPPH inhibition of 78.97 ± 0.98%, whereas the ethanol extract showed a higher value of 84.00 ± 0.35%. This difference may be attributed to the selective dissolution of different groups of polyphenols by these two solvents. Depending on their polarity and hydrogen-bonding capacity, the ChCl:LA:water-derived system and aqueous ethanol can extract distinct profiles of phenolic compounds, influencing the DPPH inhibition results [18]. Both extracts were subsequently incorporated into the spinning solutions as sources of phenolic compounds to obtain functional nanofibers enriched with bioactive components.

### 3.2. Physicochemical Properties of the Electrospinning Formulations

**Figure 2:**
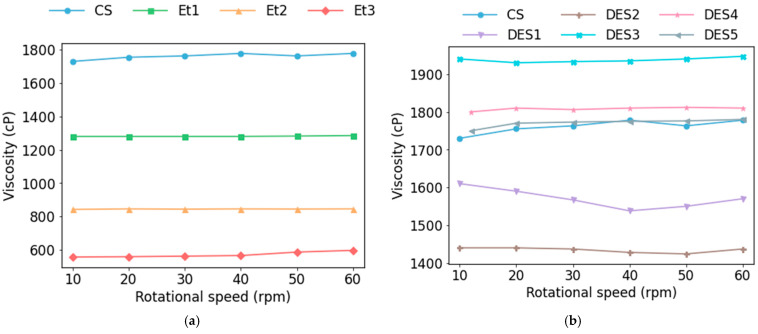
Viscosity behavior of the electrospinning formulations containing EtOH extracts (**a**) and the ChCl:LA:water-derived extracts (**b**).

Viscosity measurements were carried out to determine the flow behavior of the solutions and assess their suitability for electrospinning. The results are presented in Figure 2. For formulations containing EtOH-based extracts, a concentration-dependent decrease in viscosity was observed, with higher extract content resulting in lower viscosity. Within the investigated range (10–60 rpm), no significant variation in viscosity was observed, indicating approximately Newtonian behavior. However, these results should not be directly extrapolated to electrospinning conditions, where the solution undergoes complex deformation and jet stretching. The electrical conductivity of the spinning solutions is presented in Table S1. The CS and ethanol extract-based formulations exhibited relatively low and comparable conductivity values. In contrast, the formulations containing the ChCl:LA:water-derived extract showed a pronounced increase in conductivity, with all such samples demonstrating substantially higher values than the CS and ethanol-containing solutions. Among these samples, noticeable differences were observed, indicating that the type and composition of the extract influenced the overall ionic character of the spinning solutions. The increased conductivity may be attributed to medium-derived ionic species and other polar low-molecular-weight components, which contributed to charge transport in the solution.

### 3.3. Electrospinning of Mats

Stable electrospinning was achieved for the CS, Et1, Et2, Et3, DES1, and DES2 formulations, enabling the formation of continuous and morphologically uniform fibers under the selected processing conditions. For these systems, appropriate adjustment of the flow rate and applied voltage maintained stable jet formation throughout the electrospinning process. In contrast, the DES3, DES4, and DES5 formulations exhibited limited electrospinnability. Despite systematic optimization of the processing parameters within the investigated range, stable jet formation could not be consistently achieved, which prevented the production of homogeneous fibrous mats. These results indicate that the rheological and physicochemical properties of these solutions, particularly viscosity and conductivity, were not fully compatible with the applied electrospinning conditions.

### 3.4. Fiber Morphology

**Figure 3:**
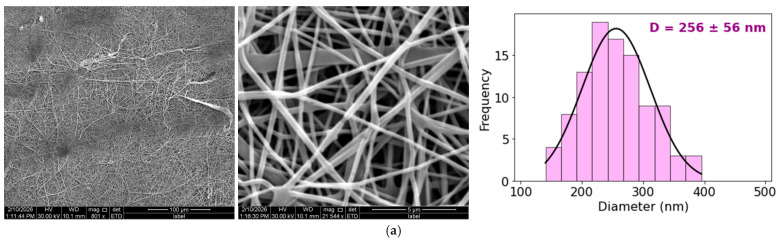

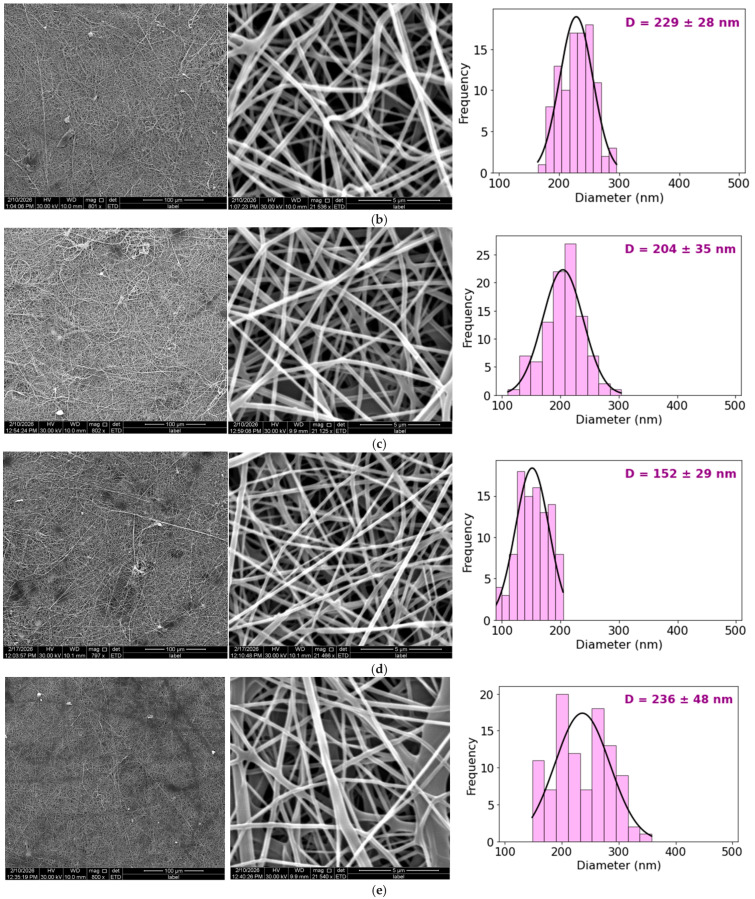

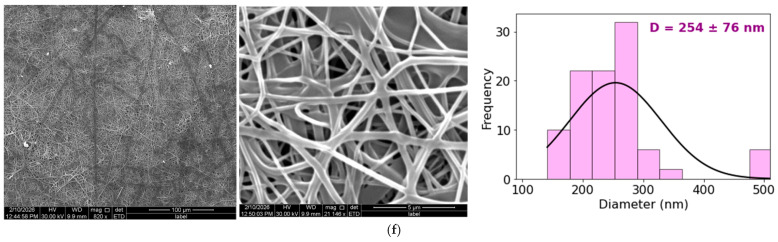
SEM images together with fiber diameter distribution for electrospun mats made from: CS (**a**), Et1 (**b**), Et2 (**c**), Et3 (**d**), DES1 (**e**), DES2 (**f**).

Figure 3 summarizes the SEM observations of the electrospun mats (CS, Et1, Et2, Et3, DES1, and DES2) and includes the corresponding histograms of fiber diameter distribution for each sample. All successfully electrospun samples exhibited predominantly continuous fibers without pronounced bead defects, although DES2 showed partial interfiber fusion and a broader diameter distribution.

A clear effect of solution composition on fiber size was observed, as the average diameters of the electrospun fibers ranged from 152 to 256 nm. The reference CS mat exhibited a mean diameter of 256 ± 56 nm. For formulations containing the ethanol extract, higher extract loading resulted in progressively thinner fibers, with the mean diameter decreasing to 229 ± 28 nm (Et1), 204 ± 35 nm (Et2), and 152 ± 29 nm (Et3). The DES-based samples displayed mean diameters of 236 ± 48 nm (DES1) and 254 ± 76 nm (DES2). DES2 exhibited the highest standard deviation, indicating greater heterogeneity in fiber size distribution. Moreover, slight interfiber connections were observed in DES2, where some fibers appeared partially fused; however, individual fibers remained distinguishable and the overall fibrous architecture was preserved. Such differences in fiber diameter and structural uniformity are expected to directly influence mass transport pathways and, consequently, the release behavior of incorporated polyphenols.

**Figure 4:**
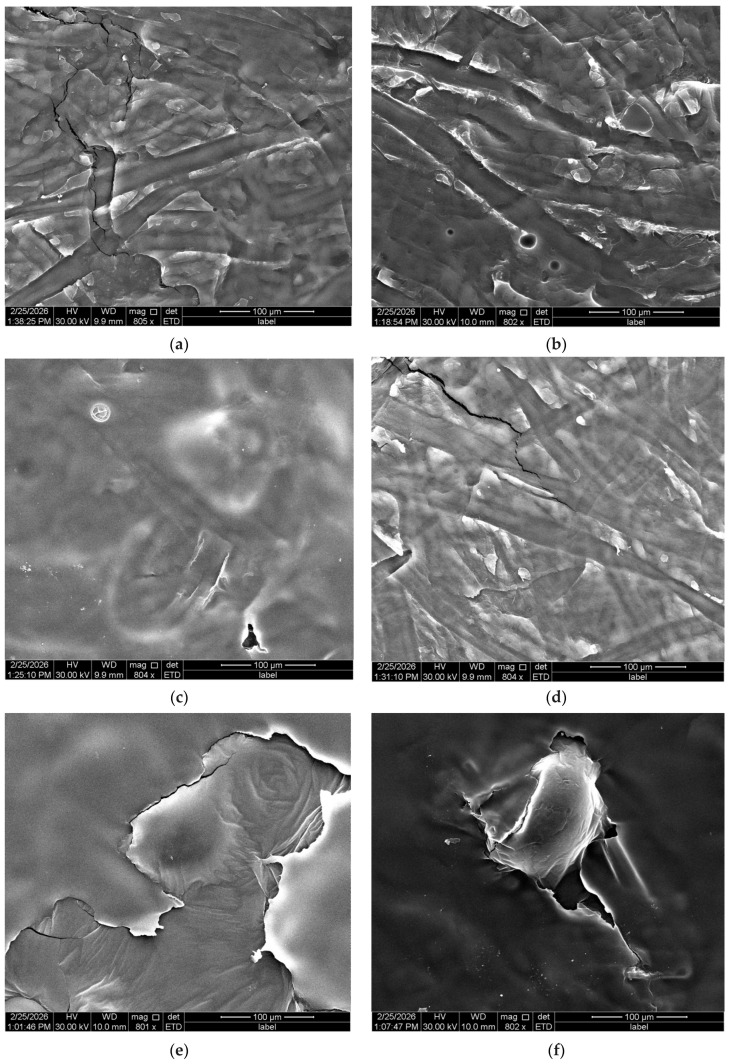

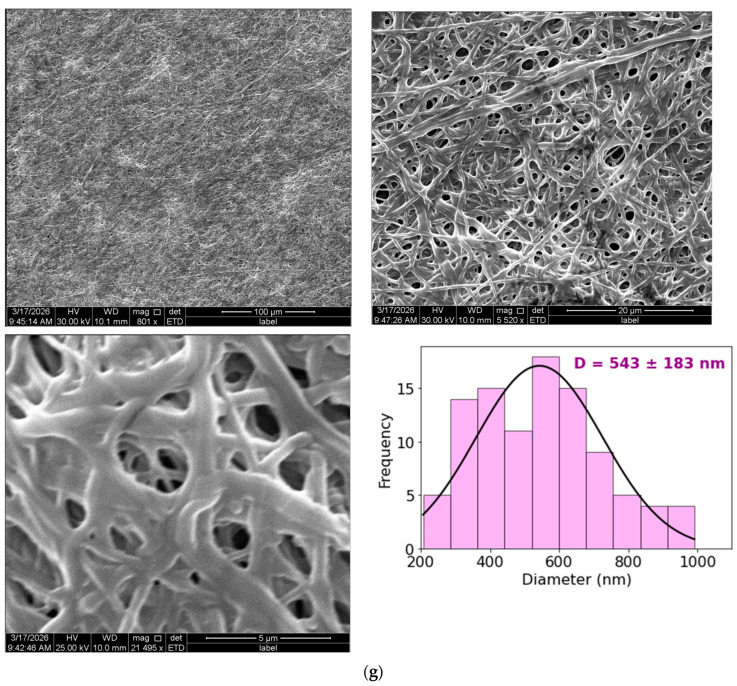
SEM pictures of crosslinked mats made from: CS (**a**), Et1 (**b**), Et2 (**c**), Et3 (**d**), DES1 (**e**), DES2 (**f**), together with SEM pictures and fiber diameter distribution of electrospun CS fibers crosslinked with 3%TA in EtOH (**g**).

After the crosslinking, the characteristic fibrous structure of all mats was no longer observed (Figure 4a–f). This structural collapse is most likely associated with the presence of water in the crosslinking medium, which may have induced swelling or partial dissolution of the hydrophilic PVA-based fibers before network stabilization. The compact, layer-like appearance observed after crosslinking should not be interpreted solely as surface deposition of the crosslinking agent, but rather as a consequence of fiber swelling and partial fusion between neighboring fibers, especially in the aqueous treatment system. The observed transformation indicates that exposure to an aqueous crosslinking environment leads to rapid water penetration into the fiber matrix, resulting in fiber coalescence and the loss of the original nanofibrous architecture. In contrast, sample CS crosslinked with 3% TA in EtOH (Figure 4g) retained its fibrous morphology, although an increase by about two times in fiber diameter was observed. This suggests that the absence of water in the crosslinking medium limited fiber swelling and prevented structural collapse. In addition, the faster evaporation of ethanol may have further reduced fiber clumping, helping to preserve their fibrous structure.

### 3.5. Mechanical Properties

#### 3.5.1. Crosslinking with an Aqueous Solution of TA

**Figure 5:**
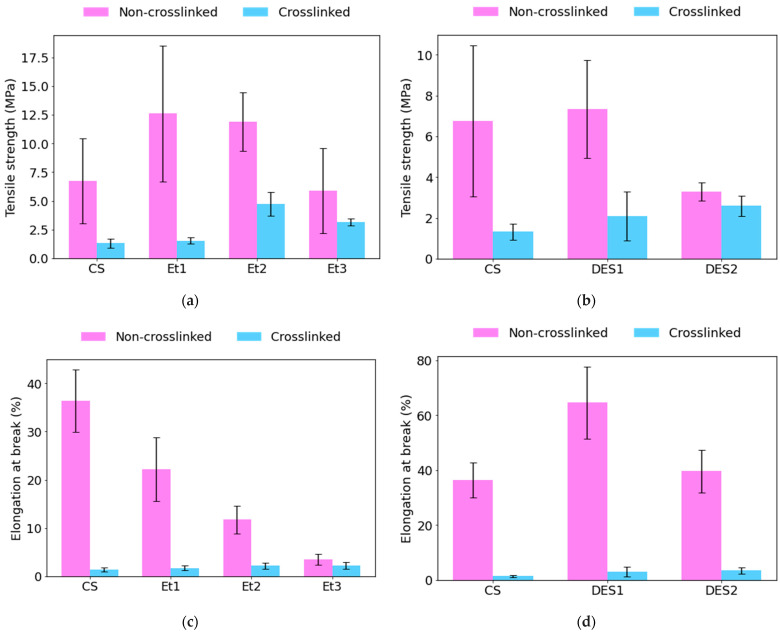
Effect of crosslinking on the mechanical properties of electrospun mats: tensile strength (**a**,**b**) and elongation at break (**c**,**d**).

Figure 5a,b present the mean tensile strength (maximum stress) values of all electrospun mats in both non-crosslinked and crosslinked states, together with the corresponding standard deviations. Among the non-crosslinked samples, DES1 and Et1 exhibited the highest tensile strength, whereas DES2 and CS demonstrated comparatively lower mechanical resistance. A general reduction in tensile strength was observed for all formulations. The decrease was particularly pronounced for Et1 and DES1, indicating that the crosslinking treatment significantly affected the mechanical integrity of these systems. In contrast, Et2 and Et3 exhibited a more moderate reduction, while CS maintained relatively low tensile strength values with limited variation. The relatively high standard deviations observed for selected non-crosslinked samples, especially DES1 and Et1, suggest structural heterogeneity within the fibrous mats. After crosslinking, the variability generally decreased, indicating a more uniform mechanical response.

Figure 5c,d illustrate the mean elongation at break values before and after crosslinking. In the non-crosslinked state, DES1 exhibited the highest elongation at break, reflecting pronounced ductile behavior. It indicates that adding the ChCl:LA:water-derived extract improved elongation properties and chain mobility compared to the control sample. The control CS sample exhibited a high level of elongation, while the addition of ethanol extract in samples Et1–Et3 progressively reduced the elongation of the fibers. These differences may arise from variations in chain mobility and intermolecular interactions among the formulations. Standard deviations were generally higher for the non-crosslinked samples, especially DES1, indicating greater variability in mechanical response. After crosslinking, elongation values became more consistent, suggesting reduced structural heterogeneity.

#### 3.5.2. Alternative Crosslinking Methods

**Figure 6:**
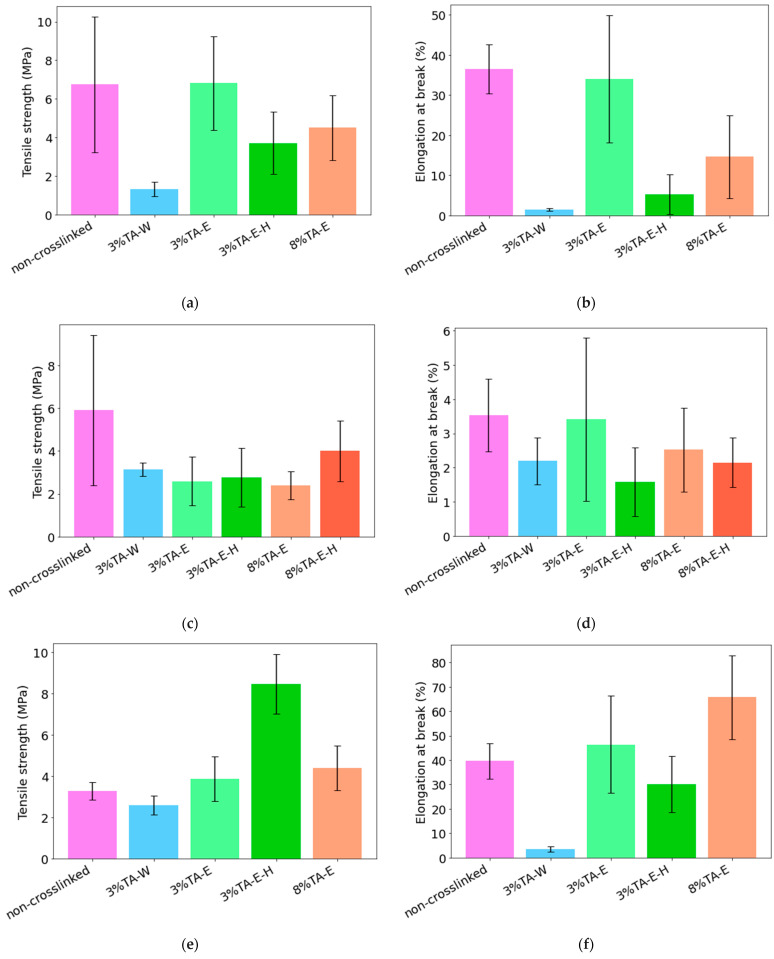
Effect of crosslinking methods on the mechanical properties of CS (**a**,**b**), Et3 (**c**,**d**), and DES2 (**e**,**f**)-based mats.

The mechanical properties of CS, Et3, and DES2 mats subjected to different crosslinking conditions are presented in Figure 6. The applied treatments included crosslinking with 3 wt% TA in water, as well as TA dissolved in ethanol at 3 wt% and 8 wt%, with or without subsequent thermal treatment. It should be noted that mechanical testing was not performed for the CS and DES2-based mats crosslinked using the 8% TA-E-H method, as these materials became too brittle after thermal treatment to be reliably handled or tested under tensile conditions. Selected crosslinked samples became brittle after treatment, particularly after thermal treatment at higher tannic acid content.

The tensile strength of the mats was strongly dependent on both the polymer matrix and the crosslinking conditions. For CS mats, crosslinking in ethanol yielded higher tensile strength than in the aqueous system, indicating more effective network formation under non-aqueous conditions. The effect of thermal treatment for the CS group indicated a significant decrease in strength compared to the non-heated sample. When the extract was added to the Et3 group, crosslinking decreased the strength in all tested groups. It is worth noting that the aqueous crosslinking solution yielded slightly better results than the ethanol-based solutions, although this difference falls within the range of statistical error. Compared to the CS group, crosslinking at elevated temperature led to improvements in mechanical strength parameters. In the DES2 group, the lowest tensile strength is observed in the non-crosslinked sample, while crosslinking with ethanol solutions results in an increase compared to the non-crosslinked sample. A significant difference compared to the CS and Et3 samples is the strong temperature dependence, resulting in approximately a twofold increase in the tensile strength of DES2 mats. The introduction of TA during the crosslinking process yields different effects depending on the material being crosslinked; it is most beneficial for the properties of mats containing NADES, indicating the validity of its application. In other cases, it generally leads to a reduction in mechanical strength. These observations indicate that crosslinking’s influence on tensile strength depends on the system’s composition. Elongation at break was more sensitive to the applied crosslinking conditions. In the case of CS mats, the highest elongation was observed for samples crosslinked with TA in ethanol at a lower concentration. Samples treated in water or subjected to additional thermal treatment exhibited a pronounced reduction in elongation, indicating increased material stiffness. Et3 mats showed consistently low elongation values across all conditions, with only minor differences between treatments, confirming the limited flexibility of these structures after crosslinking. For DES2 mats, elongation at break strongly depended on the crosslinking conditions. Samples treated with TA in ethanol, particularly at higher concentrations, showed markedly greater elongation than in the aqueous system. Thermal treatment led to intermediate values, suggesting partial reorganization of the polymer network. Selected samples also became more brittle after crosslinking, particularly after thermal treatment at higher tannic acid content, indicating excessive stiffening of the polymer network under these conditions.

### 3.6. FTIR

FTIR spectroscopy was used to evaluate the chemical structure of the obtained mats (Figure S1a–f). The broad band appearing at 3600–3000 cm^−1^ is characteristic of O-H stretching. The region of 3000–2800 cm^−1^ corresponded to CH_2_ asymmetric stretching vibrations. A peak located at 1700–1750 cm^−1^ indicated the presence of carbonyl groups. The signal observed at 1300–1000 cm^−1^ was related to C-O and C-C stretching vibrations, whereas the band in the 1000–900 cm^−1^ range was attributed to =CH_2_ wagging vibration. The bands at 850–830 cm^−1^ were assigned to C-C stretching vibrations. Additionally, the weak absorption near 600 cm^−1^ may be assigned to out-of-plane vibrations of C-H groups [19,20,21].

A broad absorption band located in the 3200–3600 cm^−1^ region was detected in nearly all spectra and was attributed to O-H stretching vibrations. This effect may result from different types of hydroxyl interactions, such as free OH groups and both intra- and intermolecular hydrogen bonds formed between hydroxyl groups and within PVA chains [22]. In general, signals around 3650 cm^−1^ are assigned to non-hydrogen-bonded hydroxyl groups, whereas bands near 3450 cm^−1^ and 3200 cm^−1^ indicate weakly and strongly hydrogen-bonded OH groups, respectively [23]. Besides the position of the OH band, the band’s shape is equally significant. Broader peaks are typically associated with more extensive intermolecular interactions and the formation of dimers or larger polysaccharide assemblies, while a narrower profile may suggest a lower contribution of intramolecular hydrogen bonding [22]. Moreover, the band at approximately 3300 cm^−1^ (O-H stretching) is also characteristic of the systems containing ChCl and LA, where hydrogen-bonding interactions between these components may occur [24]. Therefore, narrower peaks observed in the DES1 and DES2 samples may be associated with the presence of components derived from the ChCl:LA-based extraction system and their contribution to the overall hydrogen-bonding environment of the material. The difference between the CS non-crosslinked sample (3321 cm^−1^) and CS TA3–W (3260 cm^−1^), TA8–E (3273 cm^−1^), and TA8–E-H (3286 cm^−1^) samples, resulting in a broader shape, probably means more extensive intermolecular interactions, which could have been created most probably between PVA and TA. In a similar situation, a broader peak is observed in all samples after every crosslinking method. After the application of different crosslinking procedures, all samples exhibited a shift in the carbonyl-related band toward lower wavenumbers. In particular, the signal initially located at 1734 cm^−1^ moved to the 1705–1715 cm^−1^ range, depending on the crosslinking method used. Such a red shift suggests the establishment of new hydrogen-bond interactions between PVA and TA. As a consequence of these interactions, the C=O bond becomes weakened, which lowers its force constant and reduces the corresponding vibrational energy [25]. Interestingly, the most pronounced broadening and loss of a clear maximum in the O-H stretching region was observed for Et1 TA3–W, despite its lower extract content compared with Et2 and Et3 samples, while the carbonyl band still shows a red shift. This may be connected to the simultaneous presence of PVA, extract, and TA, which leads to competitive, highly heterogeneous hydrogen-bonding interactions, particularly at lower extract concentrations. This kind of competition between PVA and TA has been reported before, but given that the extract also forms hydrogen bonds, the competition could be even greater [26]. After crosslinking, the C-O stretching region (1080–1090 cm^−1^) exhibits an additional component at approximately 1020 cm^−1^ in all cases, which is connected to the TA presence in samples, which was also reported before [27,28]. The higher shift in samples crosslinked with an ethanol–TA solution may suggest stronger or more numerous hydrogen-bonding interactions than those prepared with a water–TA solution. This effect may be attributed to both the higher tannic acid concentrations (8% and 3%, respectively) and the presence of ethanol, which can modify hydrogen-bonding interactions and thus promote stronger associations between PVA and TA [29]. The FTIR results also suggest that heating does not significantly affect the chemical bonds, as reflected by the spectra of TA8–E and TA8–E-H mats. In addition, a distinct band at around 1319 cm^−1^ became more visible and can be attributed to C-O stretching vibrations associated with TA. This effect is likely connected with the higher amount of TA (8%) used as the crosslinking agent [28]. The results confirm hydrogen-bond formation between PVA and TA for all crosslinking methods, with stronger interactions observed for samples crosslinked using an 8% TA solution in ethanol.

### 3.7. Thermal Analysis

Thermal analysis of the electrospun mats was performed using TG and DTG (Figure S2). The obtained curves indicate a multi-step degradation process typical of PVA-based materials. The reference sample (PVA) exhibited a characteristic degradation profile with two dominant weight-loss stages. The initial stage was observed within the temperature interval of approximately 200–330 °C and was associated with dehydration processes and the elimination of hydroxyl groups from the polymer structure. The second and dominant degradation stage was observed between about 330 and 500 °C and corresponds to the degradation of the PVA main chain. This thermal decomposition pattern is consistent with the three-step mechanism commonly reported for PVA materials. The initial step involves the removal of physically adsorbed and chemically bound water, followed by subsequent dehydration of the polymer chains, which promotes the generation of polyene sequences. During the final stage, further chain scission and intermolecular cyclization take place, giving rise to volatile compounds together with carbonaceous residues [30]. Although the measurement was carried out up to 1040 °C, the results are presented only up to 600 °C because no significant mass changes were observed above this temperature.

Both samples containing acorn extracts exhibited similar thermal behavior. However, they left different amounts of residual mass after heating, with DES2 showing a higher residual mass than Et3, indicating limited formation of carbonaceous residues. The Et3 sample containing the ethanol extract showed a shift in the main degradation toward slightly lower temperatures, accompanied by a more intense DTG peak. In addition, this sample produced the lowest residual mass among the analyzed materials. In contrast, the DES2 sample containing the extract obtained using the ChCl:LA:water-derived system showed a different degradation pattern. Although the initial mass loss occurred at lower temperatures than in the reference samples, the residual mass at high temperatures was significantly higher. The DTG of DES2 was also broader, suggesting a more complex degradation pathway, likely influenced by medium-derived ionic and polar low-molecular-weight components present in the material.

Analysis of the TG and DTG curves indicates that all examined samples exhibit a very similar course of the main thermal degradation stage, with its maximum occurring in the range of approximately 410–430 °C (Figure S3). This suggests that neither crosslinking with tannic acid nor additional thermal treatment significantly affects the decomposition temperature of the material’s primary structure or introduces new degradation stages. The differences between the samples are quantitative and relate mainly to the course of the process. The reference sample Et3 is characterized by a more temperature-broadened degradation profile and a lower rate of mass decrease during the decomposition/degradation in relation to the other two samples. In the 8%TA-E sample, a distinctly more rapid decomposition is observed, as evidenced by a deeper, narrower DTG peak, indicating faster mass loss during the main degradation stage. For the 8%TA-E-H sample, subjected to additional thermal treatment, the DTG maximum remains within a similar temperature range; however, its intensity is lower than that of 8%TA-E and the profile is more gradual. At the same time, analysis of the TG curves reveals clear differences in the amount of solid residue, which is highest for Et3, lower for 8%TA-E-H, and lowest for 8%TA-E, suggesting that these modifications promote more complete degradation due to altered reaction pathways. The obtained results indicate that the applied modifications primarily affect the kinetics and course of degradation, without leading to a significant increase in thermal stability in terms of shifting the main decomposition effects to higher temperatures. The lack of significant changes in the temperature of the main degradation stage may result from the fact that tannic acid primarily forms physical interactions through hydrogen bonding, which do not substantially modify the thermal stability of the polymer backbone [31].

Table 3 summarizes the characteristic thermal parameters determined from the TG and DTG curves, including the temperatures corresponding to 5% and 10% mass loss (T5% and T10%), as well as the maximum degradation temperatures (Tmax_1_ and Tmax_2_) associated with individual degradation stages. High initial degradation temperatures (T5% and T10%) were observed for the reference PVA_10% sample, reflecting good thermal stability in the initial degradation region. The incorporation of the ethanolic extract (ET3) resulted in a slight decrease in these parameters, indicating an earlier onset of degradation, consistent with the presence of low-molecular-weight components in the extract. In contrast, the DES2 sample showed distinctly lower Tmax values and a single degradation maximum, reflecting its different decomposition pathway. Among the modified systems, the 8%TA-E sample exhibited the highest T5% and T10% values, indicating a delayed onset of degradation due to tannic acid crosslinking. The thermally treated 8%TA-E-H sample showed slightly lower onset temperatures than 8%TA-E, while maintaining similar Tmax values, suggesting that thermal treatment affects the early-stage degradation rather than the main decomposition step.

### 3.8. Polyphenol Release

**Figure 7:**
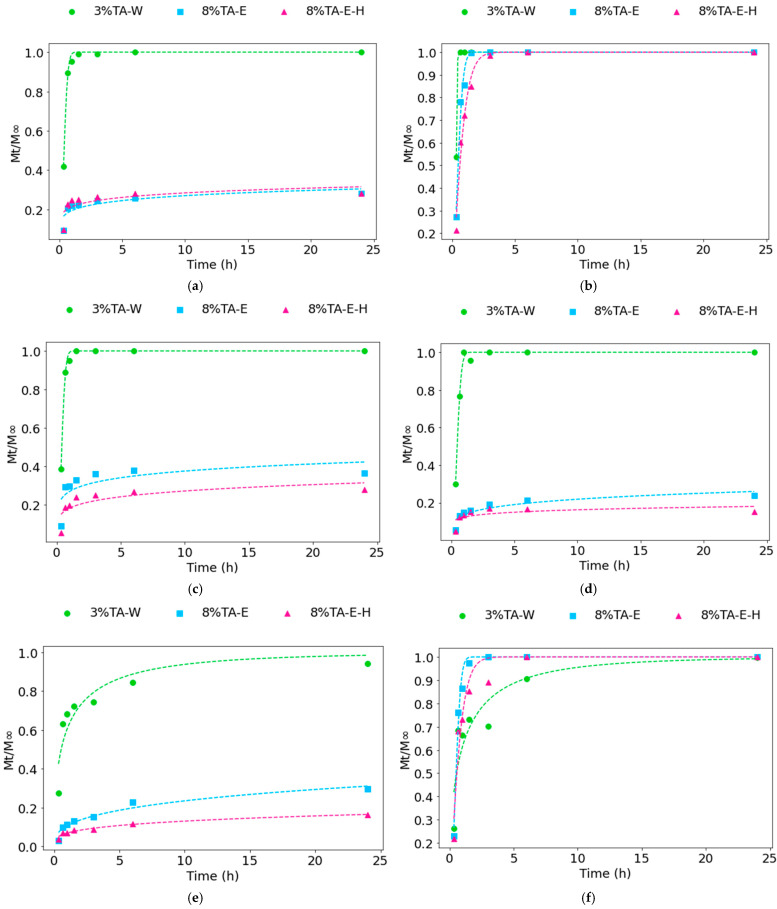

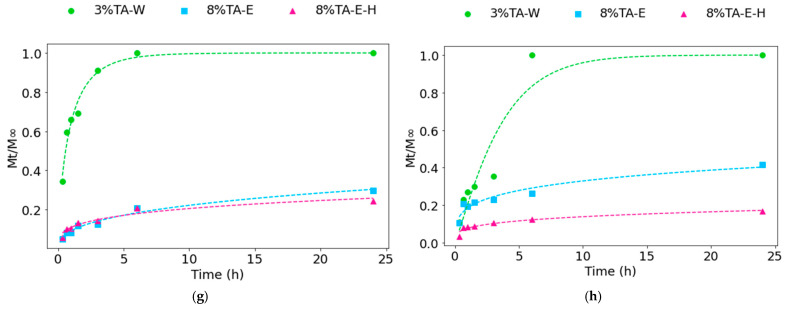
Weibull model fitting of polyphenol release profiles from electrospun mats in different media: Et3 in water (**a**), 50% EtOH (**b**), 10% EtOH (**c**), 3% acetic acid (**d**), DES 2 in water (**e**), 50% EtOH (**f**), 10% EtOH (**g**), 3% acetic acid (**h**).

The release profiles of polyphenols from Et3- and DES2-based electrospun mats showed a strong dependence on the composition of the spinning solution, the release medium, and the applied crosslinking method. The experimental data were successfully described using the Weibull, Korsmeyer–Peppas, and Peppas–Sahlin models, allowing both empirical fitting and mechanistic interpretation of the release behavior (Figure 7, Tables S2–S4). For the Et3-based mats in all media, samples crosslinked with 3% TA-W exhibited a characteristic burst release, reaching near-complete release within the initial time points. In contrast, the 8% TA-E and 8% TA-E-H systems showed slower, more controlled release in most media, accompanied by lower cumulative release, indicating stronger network stabilization and reduced diffusion. This effect was less pronounced in 50% ethanol, where all systems exhibited rapid release and reached equilibrium quickly. The influence of the release medium was clearly evident, with increasing ethanol content generally accelerating the release process. In 3% acetic acid, the release from 8% TA-E and 8% TA-E-H samples was more gradual, suggesting reduced mobility of polyphenols in this environment. DES2-based mats exhibited a markedly different release behavior. In water and 10% ethanol, the release proceeded gradually without a pronounced burst effect, indicating stronger retention of polyphenols within the matrix. Even in 50% ethanol, where the release rate increased, the profiles remained more controlled compared to the Et3 systems. Crosslinking conditions also influenced the release, with 3% TA-W samples generally showing faster release than the more densely crosslinked 8% TA-E and 8% TA-E-H variants. In acetic acid, DES2 mats exhibited the slowest release rate among all tested conditions, highlighting the combined effects of medium composition and matrix structure on diffusion. These observations indicate that release behavior is governed primarily by the interplay between fiber morphology, matrix integrity, and polymer–polyphenol interactions, all of which are strongly influenced by the extraction medium and crosslinking strategy.

The Weibull model provided a very good fit for most systems (R^2^ > 0.9), particularly for Et3-based mats crosslinked with 3% TA-W and for DES2-based systems in hydroalcoholic media (R^2^ ≈ 0.92–1.00). In contrast, poorer fits were observed for systems with limited or irregular release (e.g., Et3-acetic acid, 8% TA-E-H; R^2^ ≈ 0.41), where the scale parameter (a) exhibited unrealistically high values and large uncertainties, indicating poor parameter identifiability and reduced reliability of the model for these datasets. These cases were typically associated with very rapid release or early plateau formation, which limited the amount of informative data for fitting.

The Weibull shape parameter (β) varied across systems, reflecting differences in release profiles. Values of b < 1, mainly observed for DES2-based mats, are commonly associated with diffusion-controlled behavior, whereas b values in the range of 1–2, typical for Et3 systems, may indicate a combined diffusion–relaxation contribution. Higher b values (>2) correspond to burst release behavior followed by rapid equilibration. However, due to the empirical nature of the Weibull model and the variability in fit quality, these interpretations should be considered indicative rather than definitive.

The Korsmeyer–Peppas model was applicable only to selected systems due to its limitation to the initial release stage (Mt/M∞ ≤ 0.6). For these cases, relatively good fits were obtained (R^2^ ≈ 0.80–0.97), with release exponent values (n ≈ 0.25–0.40) suggesting predominantly diffusion-driven transport. In several systems, particularly those exhibiting rapid release, the model could not be reliably applied due to an insufficient number of data points within the valid fitting range.

The Peppas–Sahlin model provided further insight into the release mechanism by separating diffusion and relaxation contributions. In most cases, the diffusion-related parameter (k_1_) dominated, while k_2_ values were negative or close to zero, suggesting a limited contribution of polymer relaxation or potential parameter correlation effects. This behavior was particularly evident for Et3-based systems, whereas DES2-based mats exhibited more balanced contributions and m values (0.30–0.55), consistent with more gradual release profiles.

The results demonstrate that Et3-based mats promote rapid release, particularly in hydroalcoholic environments, whereas DES2-based mats provide a more sustained release profile. However, mechanistic interpretation based on model parameters should be treated with caution for datasets with lower fit quality or unstable parameters. The observed differences can be attributed to variations in matrix–polyphenol interactions and network density induced by both extract type and crosslinking method. The combined application of Weibull, Korsmeyer–Peppas, and Peppas–Sahlin models indicates that polyphenol release is primarily diffusion-driven, with a secondary contribution from polymer relaxation, more pronounced in DES2 systems.

### 3.9. Antibacterial Properties

The evaluation of the antimicrobial potential of the electrospun mats revealed a clear distinction between the neat polymer matrix and the modified fibers (Table S5). For the control samples (CS), no inhibition zones were observed against any of the tested bacterial strains. This confirms that the base-polymer mat, while structurally stable, lacks intrinsic antibacterial properties under the experimental conditions. Antibacterial activity against *S. aureus* was observed for Et3 mats and for selected ethanol–TA-crosslinked Et3- and DES2-based mats. This observation is consistent with previous findings indicating that extract-containing systems may exhibit antimicrobial properties [12]; however, in the present study, antibacterial activity was observed only for selected crosslinked extract-loaded mats. In the case of Et3, the observed activity is not unexpected, as ethanol-based extraction typically yields phenolic-rich extracts with well-documented antimicrobial properties [32]. No inhibitory effect was recorded for *Escherichia coli* or *Pseudomonas aeruginosa*. This can be attributed to fundamental differences in cell envelope structure between Gram-positive and Gram-negative strains. The outer membrane characteristic of Gram-negative bacteria constitutes an additional permeability barrier, limiting the penetration of phenolic compounds, whereas Gram-positive bacteria, such as *S. aureus*, are more sensitive due to the absence of this barrier [3]. Additionally, the amount of extract in the electrospun mat appears sufficient to inhibit *S. aureus*. However, the amount of active compounds released into the surrounding medium may remain insufficient to inhibit Gram-negative strains, including *E. coli* and *P. aeruginosa*, which possess stronger outer membrane defenses.

## 4. Discussion

The viscosity of electrospinning solutions strongly influences polymer chain entanglement, jet stability, and the resulting fiber morphology in PVA-based systems. In the present study, the reference PVA solution (CS) exhibited a viscosity of approximately 17 cP. As ethanol extract content increased, solution viscosity decreased, likely due to the presence of low-molecular-weight constituents and disruption of PVA-PVA hydrogen bonding. This viscosity reduction coincided with a clear decrease in mean fiber diameter across Et1-Et3, supporting a morphology–rheology linkage. Viscosity values in the range of 1–20 P have often been reported as favorable for electrospinning and, in some systems, for the formation of uniform, bead-free fibers [33]; however, the optimal viscosity range is strongly system-dependent. The decrease in viscosity observed in ethanol extract-containing formulations was accompanied by a reduction in fiber diameter, consistent with previous reports of a positive correlation between solution viscosity and fiber diameter in PVA electrospinning systems [34]. In contrast, the formulations containing the ChCl:LA:water-derived extract exhibited viscosities comparable to the reference solution. Components derived from this extraction medium may act as plasticizing agents depending on their composition and interactions with polymer functional groups, which can alter the viscoelastic behavior of the solution [22]. Such interactions may influence jet dynamics and fiber formation beyond what can be predicted solely from bulk viscosity measurements. Thus, the investigated solutions had viscosity values suitable for stable jet formation and the production of defect-free nanofibers. Electrospinning also depends on electrical conductivity, which affects jet stretching and fiber formation, influencing fiber diameter and morphology [35]. The low conductivity of the CS and ethanol extract-based solutions is consistent with the expected behavior of PVA solutions in aqueous or mildly polar organic media, which typically exhibit weak ionic character. In contrast, formulations containing the ChCl:LA:water-derived extract exhibited much higher conductivity, likely due to the presence of medium-derived ionic and polar low-molecular-weight components. Such an increase in conductivity may enhance jet stretching but, when excessive, can destabilize the electrospinning process [6]. In the present study, this was reflected in reduced processability and the absence of a corresponding decrease in fiber diameter, suggesting that ionic effects outweighed viscosity-related thinning under these conditions [36].

Interestingly, the higher TPC of the ChCl:LA:water-derived extract did not translate into higher DPPH inhibition. This suggests that antioxidant activity depended not only on the total amount of phenolics but also on their qualitative composition and redox reactivity, which may also contribute to differences in their interactions with the PVA matrix. The observed morphological trends for the CS and ethanol extract-based mats (Et1–Et3) are consistent with the typical behavior of PVA-based electrospinning systems. Ethanol-extract mats showed a monotonic decrease in mean fiber diameter with increasing extract content, whereas DES1-DES2 fibers remained closer to the reference diameter range but displayed broader size distributions. DES1 and DES2 fibers remained closer to the reference diameter range, but DES2 showed a broader size distribution and partial interfiber fusion, suggesting slower solidification or plasticization effects. This may influence effective porosity, mechanical integrity, and mass-transport pathways relevant to release [37]. Similar effects have been reported for PVA/DES systems, where the presence of DES led to thicker fibers and increased fiber adhesion [36], consistent with the observations in this study. Given these differences in fiber morphology and structural integrity, the stability of the electrospun mats during post-processing is a critical factor, particularly during crosslinking.

Crosslinking in an aqueous TA solution resulted in a complete loss of the characteristic fibrous morphology, as evidenced by SEM analysis. This structural collapse is attributed to PVA’s hydrophilic nature, which rapidly swells or partially dissolves upon exposure to water before effective network formation. As a consequence, fiber coalescence occurred, leading to the loss of the nanofibrous architecture and the formation of unstable, poorly defined structures. Due to these limitations, an alternative crosslinking approach based on ethanolic TA solutions was proposed. In contrast to water, ethanol acts as a non-solvent for PVA, thereby preserving the fibrous morphology during the crosslinking process and enabling more effective structural stabilization. This interpretation is supported by SEM observations showing that CS crosslinked with TA in ethanol retained a fibrous morphology, although with increased fiber diameter, indicating surface wetting and partial swelling without significant structural collapse. The structural modifications are expected to influence the morphology and the thermal stability of the materials.

The observed decrease in thermal stability for the Et3 sample may differ from reports describing improved thermal stability of PVA/polyphenol systems. For example, He and Zhang (2023) reported that incorporating pure tea polyphenols into PVA can increase the degradation temperature as a result of strong hydrogen-bond interactions between the polyphenol molecules and the polymer chains [38]. In this work, the ethanol extract constituted a complex mixture of compounds rather than a single polyphenol. The presence of additional low-molecular-weight components may disrupt the hydrogen-bonding network of PVA and alter the thermal response of the electrospun fibers.

A different effect is observed for the DES2 sample. Despite the earlier onset of low-temperature mass loss, the significantly higher residual mass indicates that the ChCl:LA:water-derived extract modifies the degradation pathway of the PVA matrix. The initial mass loss may be associated with the thermal decomposition of medium-derived ionic and polar low-molecular-weight components, such as choline chloride- and lactic acid-derived species, which can decompose at lower temperatures than the polymer matrix itself. Similar behavior has been reported for polymer systems containing deep eutectic solvents, where the decomposition of DES components introduces an additional degradation step at low temperatures [39]. At the same time, the presence of highly polar components such as choline chloride and lactic acid may promote additional intermolecular interactions with the polymer chains, influencing the thermal degradation mechanism and the formation of carbonaceous residues during decomposition. These differences in degradation behavior can be further interpreted in terms of changes in intermolecular interactions within the polymer matrix. This interpretation is consistent with recent studies on ChCl:LA-water systems, which showed that increasing water content leads to a gradual transition from water-in-DES to DES-in-water and, beyond a critical hydration threshold, to a more aqueous-like solvation environment rather than a fully preserved eutectic structure [40]. At the same time, such systems may still exhibit distinct physicochemical behavior relevant to both extraction and material formation, because compositional changes can reorganize the medium and alter its interactions with solutes. In this context, the behavior observed for the ChCl:LA:water-derived extract in the present study is better explained by the presence of medium-derived ionic and polar components and their interactions with PVA chains and extracted polyphenols, rather than by the persistence of an intact NADES network in the final material.

This interpretation is consistent with the FTIR results, which indicate changes in the hydrogen-bonding environment of the polymer matrix, and with SEM observations showing differences in fiber morphology across extract types. The combined results suggest that the ethanol extract mainly acts as a structure-disrupting component, while the ChCl:LA:water-derived extract modifies the intermolecular environment and promotes the formation of thermally stable residues during decomposition. These effects may also contribute to the different release behavior of the incorporated polyphenols. It should be emphasized that the initial ChCl:LA:water system was further combined with water and ethanol during extraction and processing, which likely altered the original eutectic organization. As a result, the extraction medium used in this work should not be considered a classical, fully preserved deep eutectic solvent, but rather a NADES-derived multicomponent ionic/polar solvent system. The observed differences between the extraction systems were reflected in the properties of the spinning solutions and the resulting electrospun mats, but these effects should not be interpreted as evidence of a preserved, highly structured eutectic network acting within the final material. Instead, the system behavior was most likely governed by medium-derived low-molecular-weight components, including ionic and polar species, and by their interactions with PVA chains and extracted polyphenols. Accordingly, the discussion was focused on composition-dependent physicochemical effects, such as changes in conductivity, thermal degradation, and release behavior, rather than on specific supramolecular effects attributed to an intact NADES structure.

Release behavior reflects the combined effects of PVA hydrophilicity, TA-mediated network stabilization and post-treatments, and extract–matrix interactions. In contrast, DES2-based mats exhibited more gradual release profiles, consistent with stronger retention and different interaction networks [41]. In the present study, a clear dependence on the crosslinking strategy was observed. Et3-based mats crosslinked using 3% TA-W exhibited very rapid release of polyphenols, reaching high cumulative release values at early time points. This behavior is directly linked to the morphological changes observed after crosslinking. SEM analysis showed that the fibrous structure of the mats was partially disrupted upon application of aqueous TA, leading to fiber swelling, fusion, or even collapse of the nanofibrous architecture. Such structural changes reduce the integrity of the electrospun network and facilitate rapid penetration of the release medium, resulting in uncontrolled and accelerated release of polyphenols. This observation is consistent with previous reports on PVA behavior in aqueous environments, where strong interactions between hydroxyl groups and water molecules lead to swelling and the loss of structural integrity [42]. In electrospun systems, insufficient stabilization of PVA fibers may result in rapid disintegration of the fibrous network, leading to a release process dominated by dissolution and diffusion rather than controlled transport.

The analysis of the applied release models consistently indicates clear differences in release behavior between the studied systems. Although the Weibull model effectively described the overall release profiles, its empirical nature limits direct mechanistic interpretation. The observed variation in the shape parameter (β) suggests that DES2-based mats follow predominantly diffusion-controlled release, while Et3 systems exhibit faster release with an additional contribution of matrix-related effects, particularly in the initial stage. These observations are supported by the limited applicability of the Korsmeyer–Peppas model, which can be used only for selected systems due to the rapid release observed in Et3-based materials. Where applicable, the model indicates diffusion-driven transport [26], confirming that diffusion is the primary mechanism governing polyphenol release. Further insight is provided by the Peppas–Sahlin model, which shows that diffusion dominates in most systems (k_1_ > k_2_) [16], while the contribution of polymer relaxation remains secondary. This effect is particularly noticeable in Et3-based mats, where release is rapid and largely controlled by diffusion. In contrast, DES2-based systems exhibit a more balanced contribution of both mechanisms, consistent with their more gradual and controlled release behavior.

Besides acting as a crosslinking agent, TA, a polyphenolic compound, may also participate in competitive intermolecular interactions with polyphenolic compounds from the *Quercus robur* extracts. Such interactions can lead to a redistribution of the interaction network, potentially affecting structural organization and release behavior. The competition between hydrogen bonding among PVA chains and PVA-TA interactions may limit hydrogen bonding between PVA and TA, therefore changing the structure of the system by decreasing PVA crystallinity [43]. As polyphenolic compounds from acorn extract also create hydrogen bonds with PVA, we assume that similar competition may also occur between these compounds and TA, reducing the effective binding of the extract’s components. The PVA–polyphenol interactions may lead to a stronger retention of these compounds within the PVA chains, thus reducing the amount of polyphenolic compounds available to be effectively released into the solvent during the release tests [44]. This competitive behavior can significantly affect the final structure and properties of the systems.

The antibacterial activity of the developed materials further confirms their functional potential. The lack of activity in control samples indicates that PVA itself lacks antimicrobial properties, whereas the observed inhibition of *Staphylococcus aureus* demonstrates the effectiveness of the incorporated polyphenols. The lack of activity against Gram-negative bacteria can be explained by the outer membrane, which limits the penetration of phenolic compounds. This behavior is consistent with previous studies that report a higher susceptibility of Gram-positive bacteria to plant-derived polyphenols [45]. Moreover, the dependence of antibacterial activity on crosslinking conditions suggests that the release and availability of active compounds are key determinants of biological performance.

It should be noted that only one NADES-derived solvent composition, based on ChCl:LA:water, was investigated in this study. Since the physicochemical properties of such systems strongly depend on their composition, including polarity, viscosity, and hydrogen-bonding capacity, the observed effects on solution behavior, fiber formation, polymer–extract interactions, and release profiles should be interpreted as specific to the system studied here. Therefore, broader conclusions regarding NADES-type systems require further comparative studies involving different solvent formulations.

## 5. Conclusions

The results demonstrate that the extraction medium plays a fundamental role not only as a source of bioactive compounds but also as a key factor governing the structure and performance of electrospun PVA materials containing *Quercus robur* extracts. The differences between extract types were also reflected in the physicochemical properties of electrospinning solutions, particularly viscosity and electrical conductivity, which influenced process stability and fiber formation. Ethanol-based extracts promoted the formation of thinner fibers, whereas formulations containing the ChCl:LA:water-derived extract showed higher conductivity and reduced electrospinning stability. These findings indicate that the investigated ChCl:LA:water-derived system may act as a functional component of the material, affecting its physicochemical behavior beyond its role as an extraction medium. The crosslinking strategy was identified as a critical parameter determining the structural integrity of the materials. Crosslinking under aqueous conditions led to the collapse of the fibrous architecture, whereas ethanol-based systems preserved the nanofibrous structure and provided more effective stabilization. The results confirm that the properties of electrospun systems may be optimized by selecting the extraction medium and the type of crosslinking treatment. Additionally, the materials exhibited selective antibacterial activity against *Staphylococcus aureus*, confirming their potential as functional components in active packaging systems. Future work should focus on optimizing crosslinking methods, particularly those involving thermal treatment, and on examining a broader range of solvent compositions related to the system investigated here, which may enable more precise control over polymer–extract interactions and the resulting functional properties of the materials.

## 6. Patents

The results presented in this work are the subject of a Polish patent application no. P.455260.

## Figures and Tables

**Table 1:** Composition of PVA-based mixtures containing different plant extracts and ethanol.

Sample ID	Extract Type	Extract Concentration (*w*/*w*)	EtOH Addition (*w*/*w*)
CS	-	-	-
Et1	EtOH	14.3%	-
Et2		28.6%	-
Et3		42.8%	-
DES1	NADES	3%	5%
DES2		5%	5%
DES3		7.5%	5%
DES4		10%	5%
DES5		10%	10%

**Table 2:** Electrospinning parameters for individual sample formulations.

Sample ID	Flow Rate (mL/h)	Voltage on Needle (kV)	Voltage on Collector (kV)	Time (min)
CS	13	18	−24	41.5
Et1	15	17	−21	36
Et2	16.5	16	−21	32
Et3	18	14	−21	30
DES1	7	18	−28	75
DES2	6	17	−30	88

**Table 3:** Key thermal decomposition temperatures obtained from TGA and DTG analysis.

Sample	T5% [°C]	T10% [°C]	Tmax_1_ [°C]	Tmax_2_ [°C]	T50% [°C]
DES2	183	248	270	—	396
ET3	247	266	285	435	359
PVA10%	263	292	300	455	375
8%TA-E	283	297	305	445	369
8%TA-E-H	226	247	295	440	354

## Data Availability

The data presented in this study are available on request from the corresponding author.

## Supplementary Materials

The following supporting information can be downloaded at: https://www.mdpi.com/article/10.3390/ma19091730/s1, Table S1: Electrical conductivity of spinning solutions; Table S2: Weibull model parameters (a,b) and coefficient of determination (R^2^) for release profiles; Table S3: Korsmeyer–Peppas model parameters (a,b) and coefficient of determination (R^2^) for release profiles; Table S4: Peppas–Sahlin model parameters ($k_{1},\;\;k_{2},\;\;m$) and coefficient of determination (R^2^) for release profiles; Table S5: Antibacterial activity of electrospun PVA mats containing Quercus robur extracts; Figure S1: FTIR spectra of nanofiber mats before and after crosslinking of CS (a), Et1 (b), Et2 (c), Et3 (d), DES1 (e), and DES2 (f) samples. Spectra in each panel have been offset vertically for clarity; Figure S2: Thermal analysis of electrospun mats: (a) TG curves and (b) DTG curves obtained for reference PVA mats and fibers containing ethanol extract (Et3) and NADES-derived extract (DES2); Figure S3: Thermal analysis of Et3-based mats crosslinked via different methods: (a) TG profiles and (b) DTG profiles.

## Abbreviations

The following abbreviations are used in this manuscript:

CFU	colony-forming units
ChCl	choline chloride
CS	control sample
DES	deep eutectic solvent
DES1–DES5	samples containing extract obtained using the ChCl:LA:water-derived system
DSC	differential scanning calorimetry
DTG	derivative thermogravimetry
Et1, Et2, Et3	samples with ethanol extract
EtOH	ethanol
FTIR	Fourier-transform infrared spectroscopy
GAE	gallic acid equivalents
LA	lactic acid
LB	Luria–Bertani medium
MS	mass spectrometry
NADES	natural deep eutectic solvent
OD600	optical density at 600 nm
PVA	poly(vinyl alcohol)
SEM	scanning electron microscopy
TA	tannic acid
TA-E	tannic acid in ethanol
TA-E-H	tannic acid in ethanol with heat treatment
TA-W	tannic acid in water
TGA	thermogravimetric analysis
TPC	total phenolic content
