# Supplementary material for: The Effect of the Extraction Medium (A Natural Deep Eutectic Solvent-Derived System vs. Ethanol) on the Properties of Electrospun PVA Fibers Containing Quercus robur Extracts
Source: Materials (Basel). 2026 Apr 24;19(9):1730. doi: 10.3390/ma19091730 (PMC13164997; doi:10.3390/ma19091730)
Supplement: Supplementary file 1 [file materials-19-01730-s001.zip › materials-4261891-supplementary.pdf]

The Effect of the Extraction Medium (A Natural Deep Eutectic Solvent-Derived System vs. Ethanol) on the Properties of Electrospun PVA Fibers Containing *Quercus robur* Extracts

Julia Wnękowicz [1], Daniel Szopa [2], Paulina Wróbel [2,*], Julia Zwolińska [2], Maciej Kaniewski [2], Jacek Chęcmanowski [3] and Anna Witek-Krowiak [2,*]

[1] Faculty of Chemistry, Wroclaw University of Science and Technology, 50-370 Wroclaw, Poland
[2] Department of Engineering and Technology of Chemical Processes, Faculty of Chemistry, Wrocław University of Science and Technology, Gdańska 7/9, 50-344 Wrocław, Poland
[3] Department of Advanced Material Technologies, Faculty of Chemistry, Wroclaw University of Science and Technology, Smoluchowskiego 25, 50-370 Wroclaw, Poland
* Correspondence: paulina.wrobel@pwr.edu.pl (P.W.); anna.witek@pwr.edu.pl (A.W.-K.)

Table S1. Electrical conductivity of spinning solutions

Sample ID	Conductivity (mS/cm)
**CS**	0.48 ± 0.02
**Et1**	0.48 ± 0.01
**Et2**	0.39 ± 0.01
**Et3**	0.39 ± 0.01
**DES1**	3.78 ± 0.02
**DES2**	5.63 ± 0.10
**DES3**	7.21 ± 0.05
**DES4**	9.49 ± 0.06
**DES5**	8.06 ± 0.07

Table S2. Weibull model parameters (a, b) and coefficient of determination (R^2) for release profiles.

Mat formulation	Environment	Crosslinking method	*a* (h)	*b* (-)	R^2
Et3	Water	3%TA-W	0.45 ± 0.01	1.98 ± 0.13	0.994
		8%TA-E	$(1.2 \pm 3.3) \times 10^4$	0.16 ± 0.05	0.665
		8%TA-E-H	$(2.2 \pm 8.7) \times 10^4$	0.14 ± 0.06	0.517
	50% EtOH	3%TA-W	0.35 ± nan	4.97 ± nan	1.000
		8%TA-E	0.58 ± 0.03	1.81 ± 0.24	0.977
		8%TA-E-H	0.82 ± 0.05	1.29 ± 0.16	0.981
	10% EtOH	3%TA-W	0.47 ± 0.01	2.11 ± 0.14	0.994
		8%TA-E	$(7.2 \pm 19) \times 10^2$	0.18 ± 0.08	0.501
		8%TA-E-H	$(3.3 \pm 8.8) \times 10^3$	0.20 ± 0.08	0.607
	Acetic acid	3%TA-W	0.55 ± 0.01	2.12 ± 0.16	0.993
		8%TA-E	$(4.8 \pm 7.7) \times 10^3$	0.23 ± 0.05	0.813
		8%TA-E-H	$(6.0 \pm 49) \times 10^6$	0.13 ± 0.07	0.415

DES2	Water	3%TA-W	1.2 ± 0.3	0.47 ± 0.13	0.831
		8%TA-E	(3.3 ± 1.7) × 10^2	0.38 ± 0.05	0.938
		8%TA-E-H	(8.2 ± 5.3) × 10^3	0.29 ± 0.03	0.964
	50% EtOH	3%TA-W	1.1 ± 0.3	0.51 ± 0.17	0.810
		8%TA-E	0.60 ± 0.03	1.99 ± 0.27	0.979
		8%TA-E-H	0.77 ± 0.08	1.22 ± 0.28	0.927
	10% EtOH	3%TA-W	0.95 ± 0.07	0.73 ± 0.09	0.976
		8%TA-E	(2.7 ± 0.8) × 10^2	0.42 ± 0.03	0.976
		8%TA-E-H	(1.4 ± 1.0) × 10^3	0.30 ± 0.04	0.929
	Acetic acid	3%TA-W	3.6 ± 0.8	1.14 ± 0.32	0.899
		8%TA-E	(2.3 ± 1.3) × 10^2	0.29 ± 0.04	0.923
		8%TA-E-H	(1.1 ± 1.1) × 10^4	0.27 ± 0.03	0.926

Table S3. Korsmeyer-Peppas model parameters (a, b) and coefficient of determination (R^2) for release profiles.

Mat formulation	Environment	Crosslinking method	*k (-)*	*n (-)*	R^2
Et3	Water	3%TA-W	-	-	-
		8%TA-E	0.19 ± 0.017	0.14 ± 0.047	0.655
		8%TA-E-H	0.21 ± 0.022	0.12 ± 0.055	0.506
	50% EtOH	3%TA-W	-	-	-
		8%TA-E	-	-	-
		8%TA-E-H	-	-	-
	10% EtOH	3%TA-W	-	-	-
		8%TA-E	0.27 ± 0.034	0.14 ± 0.068	0.483
		8%TA-E-H	0.18 ± 0.024	0.17 ± 0.066	0.595
	Acetic acid	3%TA-W	-	-	-
		8%TA-E	0.14 ± 0.013	0.20 ± 0.046	0.804
		8%TA-E-H	0.12 ± 0.015	0.12 ± 0.068	0.409
DES2	Water	3%TA-W	-	-	-
		8%TA-E	0.11 ± 0.011	0.34 ± 0.043	0.930
		8%TA-E-H	0.068 ± 0.0038	0.28 ± 0.025	0.962
	50% EtOH	3%TA-W	-	-	-
		8%TA-E	-	-	-
		8%TA-E-H	-	-	-
	10% EtOH	3%TA-W	-	-	-
		8%TA-E	0.092 ± 0.0068	0.38 ± 0.030	0.972
		8%TA-E-H	0.11 ± 0.0088	0.27 ± 0.036	0.923
	Acetic acid	3%TA-W	0.24 ± 0.019	0.39 ± 0.096	0.879
		8%TA-E	0.18 ± 0.012	0.25 ± 0.031	0.926
		8%TA-E-H	0.076 ± 0.0056	0.26 ± 0.033	0.923

Table S4. Peppas-Sahlin model parameters (k_1, k_2, m) and coefficient of determination (R^2) for release profiles.

Mat formulation	Environment	Crosslinking method	k_1	k_2	m	R^2
Et3	Water	3%TA-W	1.1 ± 0.16	-0.28 ± 0.10	0.31 ± 0.10	0.624
		8%TA-E	0.25 ± 0.03	-0.052 ± 0.015	0.34 ± 0.09	0.788
		8%TA-E-H	0.27 ± 0.04	-0.060 ± 0.020	0.35 ± 0.09	0.729
	50% EtOH	3%TA-W	1.3 ± 0.17	-0.37 ± 0.12	0.26 ± 0.09	0.515
		8%TA-E	0.96 ± 0.16	-0.20 ± 0.08	0.38 ± 0.11	0.694
		8%TA-E-H	0.78 ± 0.10	-0.13 ± 0.04	0.45 ± 0.09	0.850
	10% EtOH	3%TA-W	1.1 ± 0.17	-0.27 ± 0.10	0.32 ± 0.10	0.617
		8%TA-E	0.33 ± 0.05	-0.065 ± 0.025	0.40 ± 0.10	0.737
		8%TA-E-H	0.22 ± 0.03	-0.038 ± 0.015	0.42 ± 0.11	0.787
	Acetic acid	3%TA-W	1.0 ± 0.17	-0.22 ± 0.09	0.36 ± 0.11	0.639
		8%TA-E	0.16 ± 0.02	-0.025 ± 0.006	0.42 ± 0.08	0.913
		8%TA-E-H	0.15 ± 0.02	-0.031 ± 0.010	0.40 ± 0.08	0.779
DES2	Water	3%TA-W	0.75 ± 0.09	-0.15 ± 0.04	0.35 ± 0.10	0.816
		8%TA-E	0.11 ± 0.01	-0.010 ± 0.002	0.55 ± 0.08	0.968
		8%TA-E-H	0.075 ± 0.005	-0.0079 ± 0.003	0.37 ± 0.08	0.969
	50% EtOH	3%TA-W	0.74 ± 0.10	-0.14 ± 0.04	0.35 ± 0.12	0.791
		8%TA-E	0.93 ± 0.16	-0.19 ± 0.08	0.39 ± 0.11	0.690
		8%TA-E-H	0.80 ± 0.11	-0.14 ± 0.05	0.42 ± 0.10	0.809
	10% EtOH	3%TA-W	0.75 ± 0.04	-0.13 ± 0.02	0.43 ± 0.04	0.964
		8%TA-E	0.095 ± 0.008	-0.0073 ± 0.0014	0.53 ± 0.08	0.982
		8%TA-E-H	0.12 ± 0.008	-0.014 ± 0.002	0.48 ± 0.05	0.977
	Acetic acid	3%TA-W	0.20 ± 0.06	-0.0071 ± 0.0054	0.98 ± 0.17	0.936
		8%TA-E	0.15 ± 0.71	0.031 ± 0.72	0.21 ± 0.76	0.927
		8%TA-E-H	0.086 ± 0.008	-0.011 ± 0.003	0.38 ± 0.10	0.941

Table S5. Antibacterial activity of electrospun PVA mats containing *Quercus robur* extracts.

**SAMPLE**	**Diameter of inhibition (mm)**		
	E. coli	*P. aeruginosa*	*S. aureus*
**CONTROL**	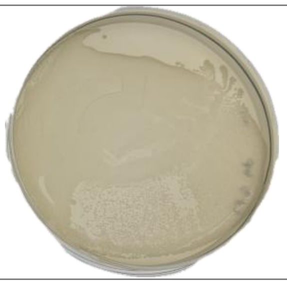	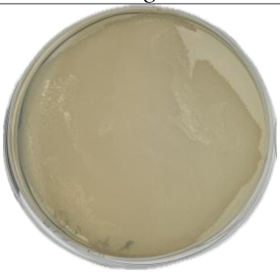	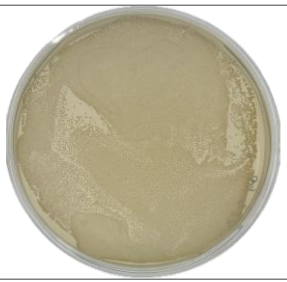
**CS**	0.0 ± 0.0	0.0 ± 0.0	0.0 ± 0.0

	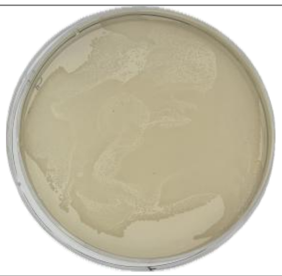	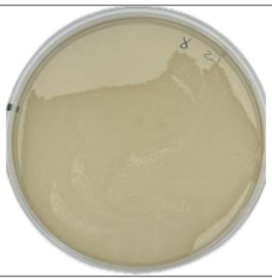	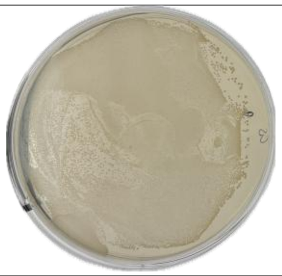
**CS-TA-E-H**	0.0 ± 0.0	0.0 ± 0.0	0.0 ± 0.0
	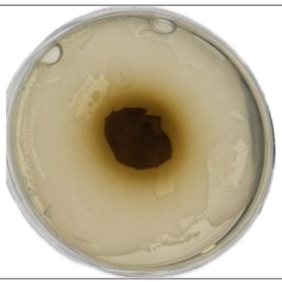	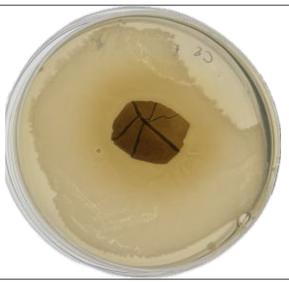	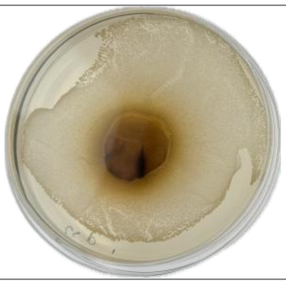
**CS-TA-E**	0.0 ± 0.0	0.0 ± 0.0	0.0 ± 0.0
	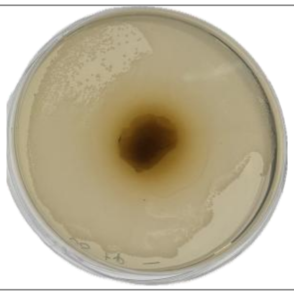	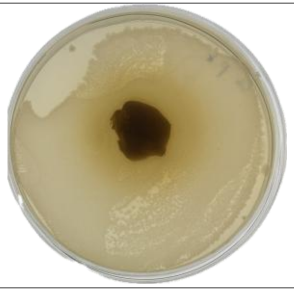	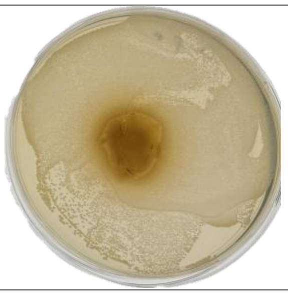
**DES2**	0.0 ± 0.0	0.0 ± 0.0	0.0 ± 0.0
	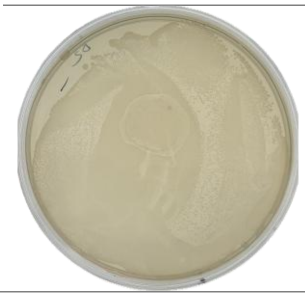	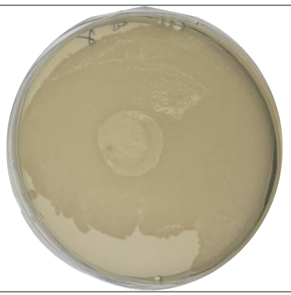	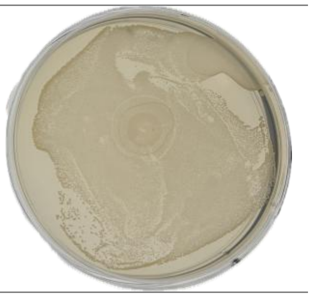
**DES2-TA-E-H**	0.0 ± 0.0	0.0 ± 0.0	20.16 ± 1.4
	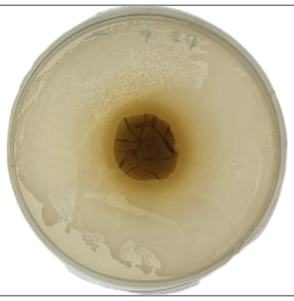	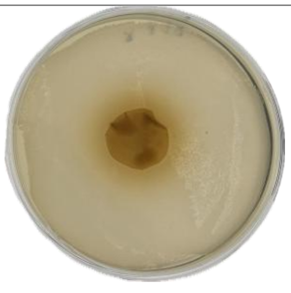	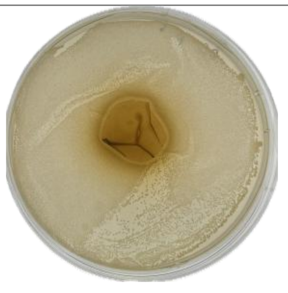
**DES2-TA-E**	0.0 ± 0.0	0.0 ± 0.0	19.29 ± 1.31

	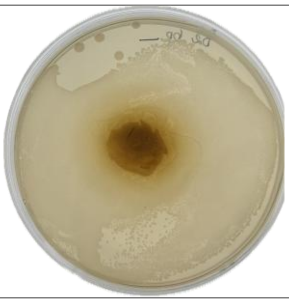	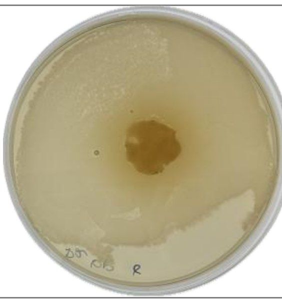	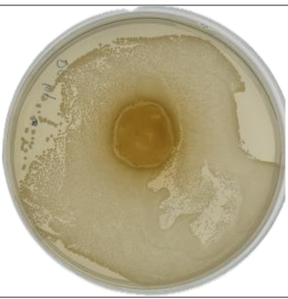
**Et3**	0.0 ± 0.0	0.0 ± 0.0	15.46 ± 0.81
	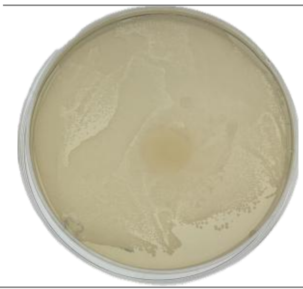	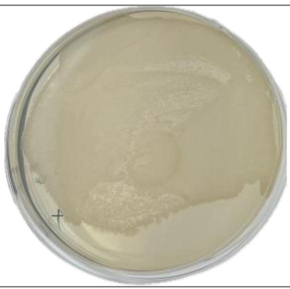	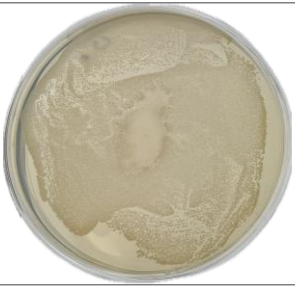
**Et3-TA-E-H**	0.0 ± 0.0	0.0 ± 0.0	20.8 ± 0.38
	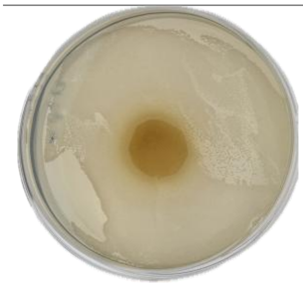	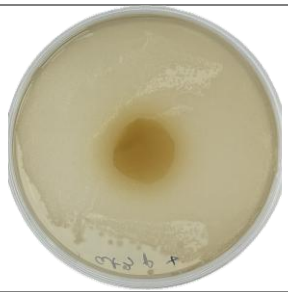	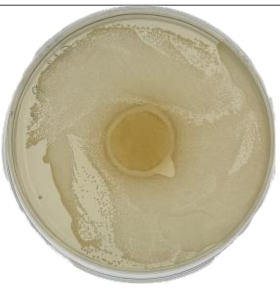
**Et3-TA-E**	0.0 ± 0.0	0.0 ± 0.0	19.69 ± 0.45
	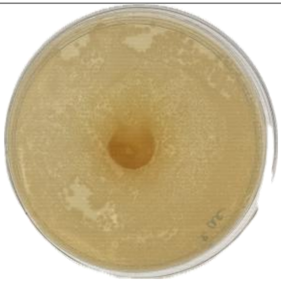	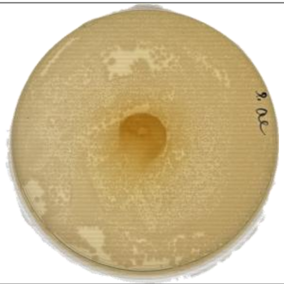	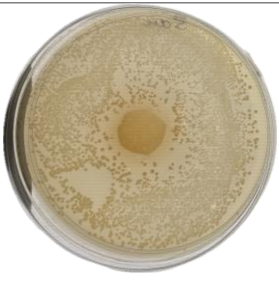

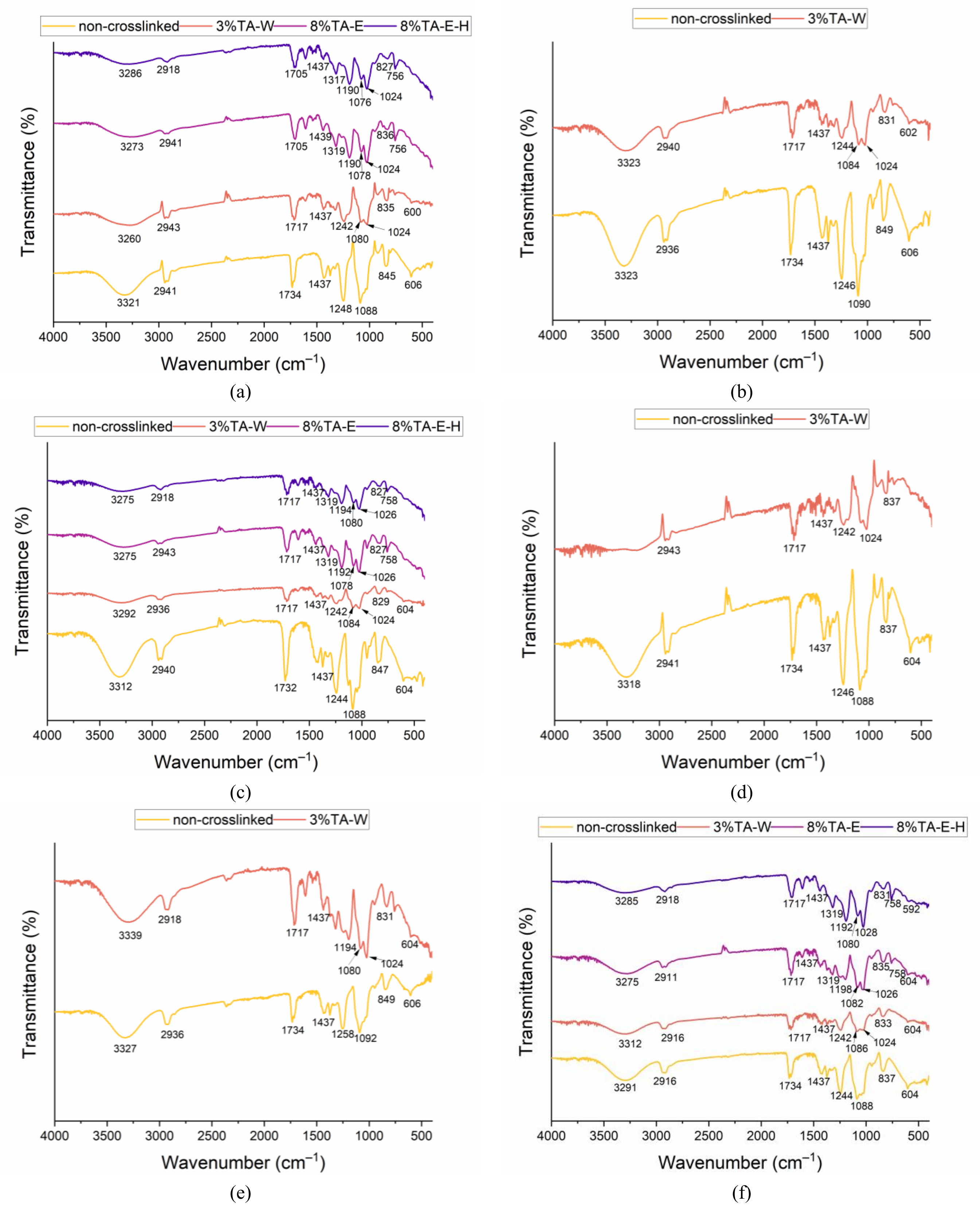

Figure S1. FTIR spectra of nanofiber mats before and after crosslinking of CS (a), Et1 (b), Et2 (c), Et3 (d), DES1 (e), and DES2 (f) samples. Spectra in each panel have been offset vertically for clarity.

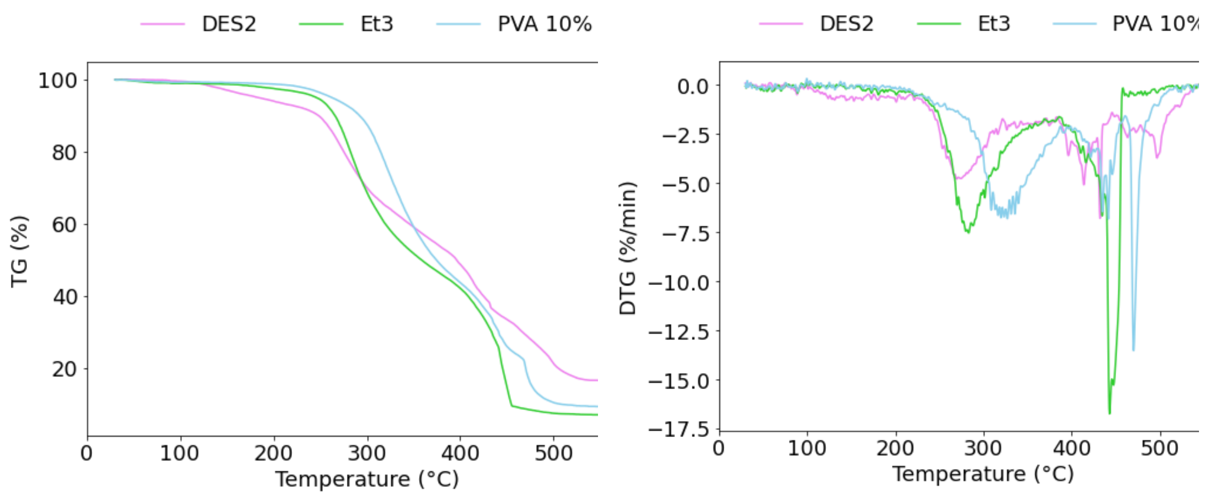

(a) (b)

Figure S2. Thermal analysis of electrospun mats: (**a**) TG curves and (**b**) DTG curves obtained for reference PVA mats and fibers containing ethanol extract (Et3) and NADES-derived extract (DES2).

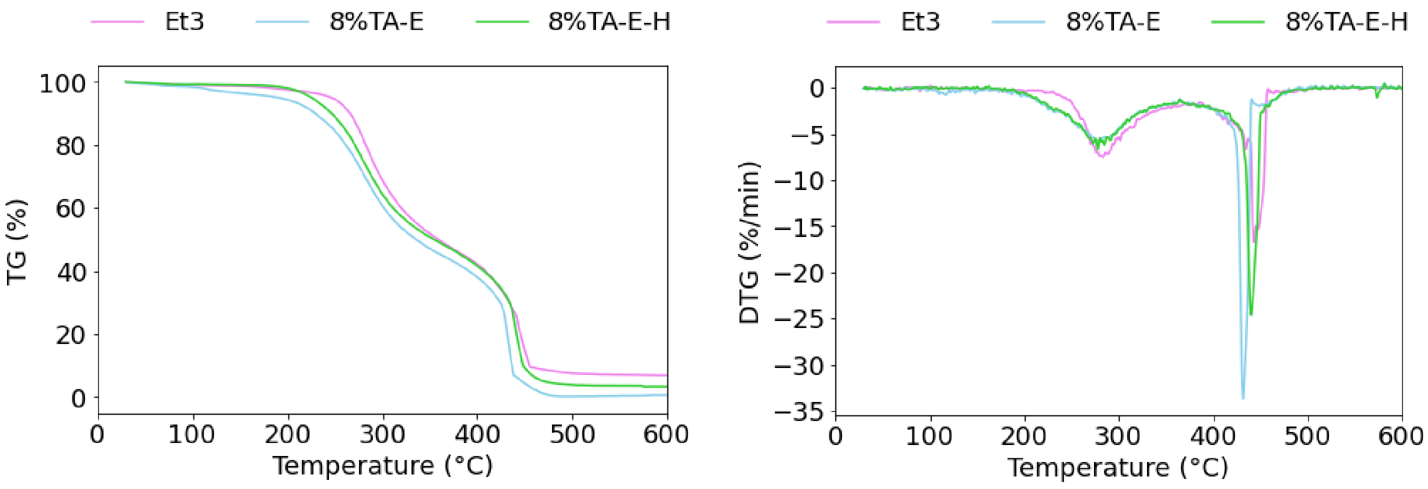

(a) (b)

Figure S3. Thermal analysis of Et3-based mats crosslinked via different methods: (a) TG profiles and (b) DTG profiles.
